# RAB1A promotes *Vaccinia virus* replication by facilitating the production of intracellular enveloped virions

**DOI:** 10.1016/j.virol.2014.11.007

**Published:** 2015-01-15

**Authors:** Tali Pechenick Jowers, Rebecca J. Featherstone, Danielle K. Reynolds, Helen K. Brown, John James, Alan Prescott, Ismar R. Haga, Philippa M. Beard

**Affiliations:** a The Roslin Institute and Royal (Dick) School of Veterinary Studies, University of Edinburgh, Roslin, Midlothian EH25 9RG, Scotland; b Division of Cell Signalling and Immunology, College of Life Sciences, University of Dundee, Dundee DD1 5EH, Scotland

**Keywords:** RAB1A, *Vaccinia virus*, Poxvirus, Golgi

## Abstract

*Vaccinia virus* (VACV) is a large double-stranded DNA virus with a complex cytoplasmic replication cycle that exploits numerous cellular proteins. This work characterises the role of a proviral cellular protein, the small GTPase RAB1A, in VACV replication. Using siRNA, we identified RAB1A as required for the production of extracellular enveloped virions (EEVs), but not intracellular mature virions (IMVs). Immunofluorescence and electron microscopy further refined the role of RAB1A as facilitating the wrapping of IMVs to become intracellular enveloped virions (IEVs). This is consistent with the known function of RAB1A in maintenance of ER to Golgi transport. VACV can therefore be added to the growing list of viruses which require RAB1A for optimal replication, highlighting this protein as a broadly proviral host factor.

## Highlights

• Characterisation of the role of the small GTPase RAB1A in VACV replication.
• RAB1A is not required for production of the primary virion form (IMV).
• RAB1A is required for production of processed virion forms (IEVs, CEVs and EEVs).
• Consistent with known role of RAB1A in ER to Golgi transport.

## Introduction

*Vaccinia virus* (VACV) is the prototypic virus of the Orthopoxvirus genus of *Poxviridae,* a family of large, double stranded DNA viruses which undertake a complex replication cycle entirely within the cytoplasm of an infected cell. Multiple forms of the poxvirus virion are produced during the cycle, and can be differentiated by their cellular location, number of membranes, abundance and function. After entering a cell, via plasma membrane fusion or endocytosis, the VACV virion travels to a perinuclear location to establish a cytoplasmic viral factory (Moss, 2007). These factories produce abundant numbers of intracellular mature virus (IMV), which consists of a core particle surrounded by a single lipid membrane that is embedded with entirely nonglycosylated viral proteins. A small fraction of IMVs (approximately 1% (Payne, 1980)) exit the viral factory and are wrapped by two additional cellular membranes that are embedded with glycosylated viral proteins to form intracellular enveloped virions (IEVs) (Hiller and Weber, 1985). IEVs then travel to the periphery of the cell where their outermost membrane fuses with the plasma membrane, leaving a cell associated virion (CEV) surrounded by the two remaining membranes. CEVs released from the surface are known as extracellular enveloped virions (EEVs). IMVs are robust virions and capable of long-term survival in the environment. In comparison CEVs and EEVs are more labile but crucial for efficient and timely cell to cell spread of VACV in vivo and in vitro (Blasco and Moss, 1992; Smith et al., 2003). Alternative nomenclature refers to IMVs as mature virions, IEVs as wrapped virions, and CEVs and EEVs as extracellular virions (Moss, 2006).

The intricate cell–virus interactions involved in poxvirus morphogenesis are still incompletely understood. High throughput, unbiased, RNA interference screens have been used to identify cellular proteins which are required for poxvirus replication (Beard et al., 2014; Mercer et al., 2012; Sivan et al., 2013; Teferi et al., 2013). Two of these screens identified RAB1A as a strongly proviral host factor (Beard et al., 2014; Sivan et al., 2013). Only a small number of individual cellular proteins were identified in multiple screens, suggesting these particular proteins play a crucial role in the virus life cycle and are therefore worthy of detailed investigation.

RAB1A is a member of the Rab GTPase protein family. This family contains over 60 human Rab proteins which localise to specific intracellular membranes and act as directors and organisers of membrane trafficking including pathways among the ER, golgi, endosomes, lysosomes, phagosomes and autophagosomes (Stenmark, 2009). The most well-known function of RAB1A is to facilitate vesicle trafficking from the endoplasmic reticulum (ER) to the Golgi. This pathway consists of the ER, the ER–Golgi intermediate compartment (ERGIC), and the cis face of the Golgi. Anterograde transport begins at specialised areas of the ER known as ER exit sites (ERES) which produce and release vesicles coated in the membrane coat complex COPII. The small GTPase Sar1 is essential for the formation of these COPII vesicles (Donaldson and Jackson, 2011). RAB1A localises predominantly to the ERGIC membrane and recruits the tethering factor p115 to the COPII coated vesicles, facilitating the formation of a fusion complex and thus directing COPII vesicles to the Golgi for delivery of their cargo (Allan et al., 2000). However, in addition to its function in ER to Golgi transport, RAB1A is also involved in early Golgi trafficking (Yamasaki et al., 2009), the motility of early endocytotic vesicles, early endosome to Golgi trafficking (Mukhopadhyay et al., 2011), regulation of the actin cytoskeleton (Kicka et al., 2011), recycling of the integrin protein ITGB1 to the cell surface (Wang et al., 2010) and autophagy (Winslow et al., 2010). RAB1A is therefore a multifunctional protein with roles in varied cellular processes.

Previous work has revealed a role for RAB1A in the life cycles of a number of viruses. RAB1A is required for the trafficking of viral envelope glycoproteins of HIV (Nachmias et al., 2012) and HSV-1 (Zenner et al., 2011), highlighting the protein׳s role in maintenance of a functional Golgi. In contrast, RAB1A plays a direct role in Hepatitis C virus replication, interacting with the viral protein NS5A and promoting lipid droplet formation (Nevo-Yassaf et al., 2012; Sklan et al., 2007). Examination of known RAB1A functions produces a number of hypotheses for the proviral role of RAB1A in VACV replication cycle. In an analogous manner to HIV and HSV-1, RAB1A may be required to maintain Golgi function which, for poxviruses, is vital for the production of IEVs and therefore CEVs and EEVs (Ulaeto et al., 1995). However, alternative hypotheses exist. RAB1A has been shown to be rapidly recruited to plasma-membrane lipid rafts within 30 min of VACV infection (Schroeder et al., 2012), suggesting a possible role in virus entry. In support of this theory, RAB1A regulates the recycling of ITGB1 to the cell surface (Wang et al., 2010) and this integrin protein has been shown to facilitate VACV entry (Izmailyan et al., 2012). An alternative hypothesis is that RAB1A plays a novel role in VACV replication. This is the case for another cellular protein concerned with vesicle trafficking, golgin-97. Golgin-97 is a tethering factor which facilitates retrograde transport from endosomes back to the trans-Golgi network (Lieu et al., 2007; Lu et al., 2004). Knockdown of golgin-97 in VACV infected cells unexpectedly resulted in disruption of IMV formation and accumulation of immature virions (Alzhanova and Hruby, 2006, 2007). It is unclear why this protein, which facilitates a late stage of the vesicle transport system, should inhibit early stages of VACV morphogenesis.

Given the multiple plausible hypotheses for the role of RAB1A in VACV replication, we investigated the stage of the viral life cycle for which RAB1A is required.

## Results

### RAB1A is a proviral cellular factor in VACV replication

**Fig. 1:**
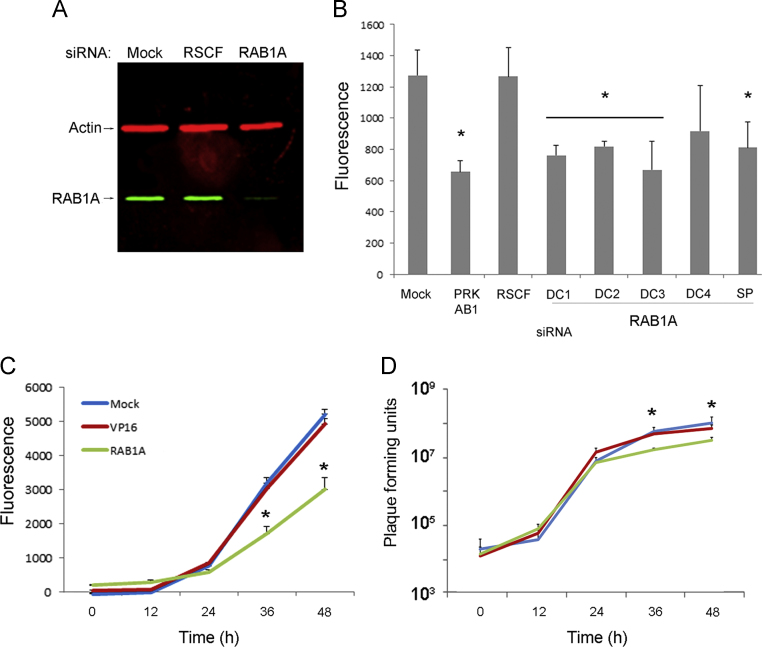
A reduction in RAB1A expression inhibits VACV replication. (A) Western blot image of protein lysates from HeLa cells mock transfected or transfected with non-targeting siRNA (RSCF) or a siRNA SMARTpool targeting RAB1A, probed with antibody against RAB1A (green) or actin (red), and visualised using the Odyssey^®^ Infrared Imaging System (Li-Cor Biosciences). (B) HeLa cells (seeded on 96 well plates) were mock transfected or transfected in triplicate with non-targeting siRNA (RSCF), or siRNA targeting PRK-AB1, a RAB1A siRNA SMARTpool (SP), or four individual deconvoluted RAB1A siRNAs (DC1–DC4). After 48 h, cells were infected with VACV-A5L-EGFP at MOI=0.1, and after a further 48 h fluorescence levels were measured. Values shown are the mean corrected for background, error bars represent standard error of the mean, and *P* values <0.05 are indicated (*t*-test). (C, D) Multistep growth curves. HeLa cells were mock transfected or transfected with non-targeting siRNA (VP16) or a siRNA SMARTpool targeting RAB1A for 48 h, then infected with a MOI of 0.1 with either (C) VACV-A5L-GFP and virus levels determined by fluorescence, or (D) VACV and the amount of infectious virus levels in both the supernatant and cells combined determined by plaque assay on BS-C-1 cells. (C) Means of three technical replicates, error bars indicating the standard deviation. *P* values <0.05 are indicated (*t*-test). The data are representative of three biological replicates. (D) Average and standard error of the mean (SEM) of five biological replicates. *P* values <0.05 are indicated (mixed model analysis).

RAB1A was identified as a strongly proviral cellular protein in two independent, high-throughput siRNA screens of VACV replication (Beard et al., 2014; Sivan et al., 2013). To investigate this result further HeLa cells were mock transfected or transfected either with a non-targeting negative control siRNA (RSCF) or siRNA targeting RAB1A. A siRNA SMARTpool containing four different siRNAs all targeting RAB1A was used to enhance the magnitude and specificity of protein knockdown. After 48 h of transfection cellular proteins were collected and the level of RAB1A protein in the cell lysates was compared using western blotting. The level of RAB1A was substantially reduced in cells treated with siRNA targeting RAB1A (Fig. 1A), confirming the efficacy of the siRNA knock down. The impact of RAB1A on VACV replication and spread was then examined using VACV-A5L-EGFP, a VACV strain which has the A5 viral capsid protein tagged with EGFP, thereby enabling virus growth to be estimated by measuring fluorescence levels (Beard et al., 2014; Carter et al., 2003). HeLa cells were mock transfected or transfected with non-targeting RSCF siRNA (negative control), siRNA targeting PRK-AB1 (positive control) which is required for efficient VACV replication (Moser et al., 2010), or the RAB1A siRNA SMARTpool. In addition, the SMARTpool was deconvoluted to the four constituent siRNAs and each tested individually. After 48 h the cells were infected with VACV-A5L-EGFP at a low MOI of 0.1 and fluorescence measured after a further 48 h, allowing multiple rounds of virus replication to have occurred (Fig. 1B). Cells were also examined using light microscopy for evidence of any cytotoxic effect of the siRNAs with none detected (data not shown). Fluorescence levels were comparable in the negative control samples (mock and RSCF transfected), but significantly reduced in the wells treated with positive control siRNA (PRK-AB1), the RAB1A SMARTpool and three of the four deconvoluted RAB1A siRNAs.

To assess the effect of RAB1A depletion on viral growth kinetics a multistep growth curve was carried out using both fluorescence levels and viral plaque titration to measure virus replication. Cells were mock transfected or transfected with siRNA targeting the herpesvirus protein VP16 (negative control) or the RAB1A siRNA SMARTpool. After 48 h cells were infected with VACV-A5L-EGFP at an MOI of 0.1 followed by measurement of fluorescence and viral titres at 12 h intervals. EGFP expression was comparable in the negative control samples (mock and VP16) but significantly lower at 36 and 48 hpi in cells with reduced levels of RAB1A (Fig. 1C). Virus titration (Fig. 1D) revealed a statistically significant reduction in the amount of infectious virus at 36 and 48 hpi in cells lacking RAB1A compared with control cells. Overall, these results substantiate the previously reported evidence from the high throughput siRNA screens that RAB1A is required for efficient multicycle growth of VACV (Beard et al., 2014; Sivan et al., 2013).

### RAB1A is required for optimal production of EEVs but not IMVs

**Fig. 2:**
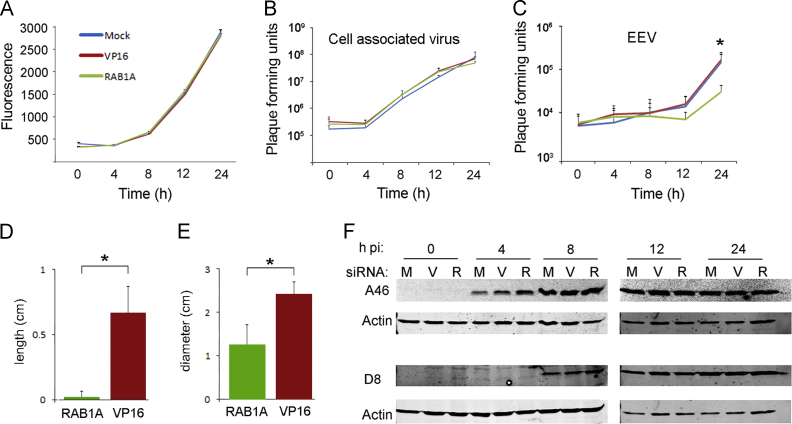
RAB1A plays a role in EEV but not IMV production. (A–C) Single-step growth curve. HeLa cells were mock transfected or transfected with non-targeting siRNA (VP16) or a siRNA SMARTpool targeting RAB1A for 48 h then infected with VACV (MOI=5). Total virus loads were determined at the indicated times by fluorescence (A) or plaque assay (B, C). (A) Mean value of six technical replicates is shown and error bars represent standard error of the mean. The results are representative of three biological replicates. Plaque assays of the cellular (B) and supernatant (C) fractions of infected cell cultures were carried out on BS-C-1 cells. The graph shows the average and standard error or the mean (SEM) of four biological replicates. *P* values <0.05 are indicated (mixed model analysis). (D) Graph comparing the length of comet tails (*n*=10) in BS-C-1 cells transfected with non-targeting VP16 siRNA or a siRNA SMARTpool targeting RAB1A for 48 h then infected with VACV. (E) Graph comparing plaque diameter (*n*=10) in BS-C-1 cells transfected with non-targeting VP16 siRNA or a siRNA SMARTpool targeting RAB1A for 48 h then infected with VACV. (F) HeLa cells were mock transfected (M) or transfected with non-targeting VP16 siRNA (V) or a siRNA SMARTpool targeting RAB1A (R) for 48 h then infected with VACV at MOI=5. Whole-cell lysates were collected at 0, 4, 8, 12, and 24 hpi. Proteins were separated by SDS-PAGE and analysed by immunoblotting with the indicated antibodies using the Odyssey^®^ Infrared Imaging System (Li-Cor Biosciences).

In order to determine the stage of the VACV life cycle for which RAB1A is required we carried out a onestep growth curve measuring fluorescence levels and viral titres at regular intervals. HeLa cells were mock transfected or transfected with siRNA targeting either VP16 or RAB1A, and after 48 h infected with VACV-A5L-EGFP (MOI=5). In contrast to the multistep growth curve, EGFP levels in RAB1A, VP16 and mock transfected cells were comparable at all time points in the onestep growth curve (Fig. 2A). EGFP expression by the VACV-A5L-EGFP virus is under the control of the A5 promoter which is expressed both early and late in the viral transcriptional cascade (Yang et al., 2011). Therefore this suggests that all stages of viral replication up to and including late gene expression are unaffected by loss of RAB1A. Titration of the cell associated fraction (which consists almost entirely of IMVs) revealed comparable virus titres in control and RAB1A siRNA transfected cells at all time points, in agreement with the uniform fluorescent results (Fig. 2B).

To examine specifically the impact of RAB1A on later stages of VACV replication we titrated the amount of virus present in the supernatant of cells with normal or reduced amount of RAB1A. Neutralising antibody to IMVs was added to the supernatant fraction prior to titration to ensure that only EEVs that had been released from the cell were titrated. We carried out a onestep growth curve four times (four biological replicates) and at 24 hpi identified an average 0.5 log_10_ reduction in the amount of virus in the supernatant fraction from cells in which RAB1A was knocked down in comparison to control cells (*P*<0.05) (Fig. 2C), indicating that fewer EEVs are produced from cells lacking normal levels of RAB1A.

The number of CEVs and EEVs produced by an infected cell influences the size and morphology of the viral plaque, and the length of the viral “comet tail” which is formed by secondary plaques emanating from a primary plaque when liquid overlay is used (Blasco and Moss, 1991, 1992). We therefore compared viral plaque size and comet tail length in BS-C-1 cells which had been transfected with nontargeting siRNA (VP16) or siRNA targeting RAB1A (Fig. 2D and E). Comet tails were significantly shorter and plaque size was significantly smaller in BS-C-1 cells treated with RAB1A targeting siRNA, supporting the results of the onestep growth curve described above which revealed a reduction in EEV production.

As outlined above, the association of RAB1A with lipid rafts and ITGB1 could suggest a role for this protein in VACV cell entry. We have previously shown that subtle defects in virus entry can be detected by delayed early gene expression (Haga et al., 2014). We therefore compared the production of A46, an early VACV protein (Stack et al., 2005), in cells with normal and reduced levels of RAB1A (Fig. 2F). A46 was first detected in all samples 4 hpi and remained present at comparable levels throughout the time course. We also measured production of the late viral protein D8 (Fig. 2F). This was first detected at 8 hpi and, as expected, was expressed at similar amounts in all treatments at all time points. These results support the onestep growth curve results which indicate that all stages of virus replication up to and including the production of the first mature virion form, the IMV, are unaffected by a reduction in the amount of RAB1A present in the cell, and that RAB1A is therefore not required for efficient VACV entry.

### RAB1A is required for production of IEVs

**Fig. 3:**
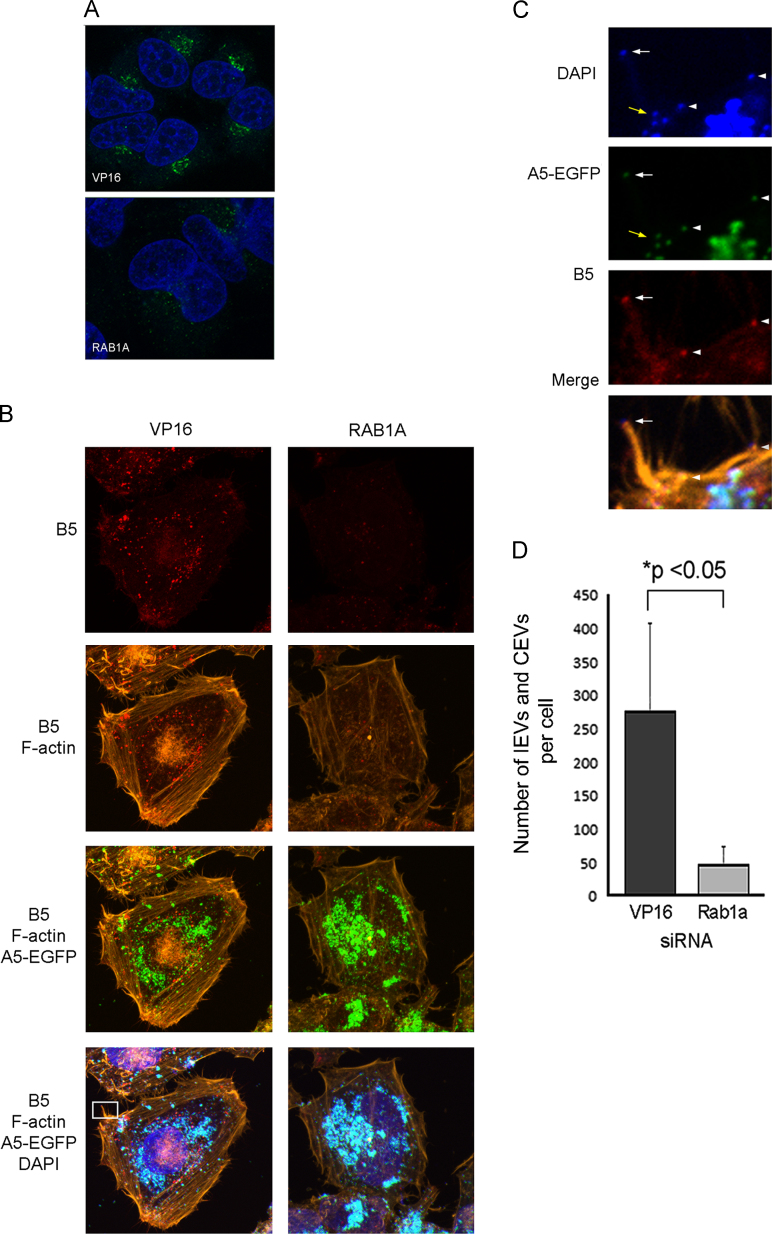
RAB1A knock down causes dispersal of the Golgi and a reduction in the number of B5-labelled virions. HeLa cells were transfected with siRNA targeting VP16 or RAB1A for 48 h then (A) infected with VACV at 5 PFU/cell for 8 h and then fixed and labelled with anti-GM130 antibody which labels the Golgi (green) and DAPI (blue). Images were acquired with a Zeiss LSM 710 confocal microscope. (B) Cells were transfected as described above then infected with VACV-A5L-EGFP (green) at 5 PFU/cell for 8 h and then fixed and labelled with anti-B5 antibody (red), phalloidin (orange), and DAPI (blue). Images are maximum intensity projections acquired with the Zeiss LSM 710 confocal microscope. The cell shown in panels on the left is representative of the population of cells transfected with nontargeting siRNA (VP16). The cell shown in panels on the right is representative of the population of cells transfected with siRNA targeting RAB1A. (C) High magnification image of the area within the white rectangle on image (B), revealing an actin tail with a CEV at the tip (white arrow), as well as a cluster of IMVs (yellow arrows) and IEV/CEVs on or near the surface of the cell (white arrowheads). (D) IEVs or CEVs (identified by red B5 staining colocalised with A5-EGFP signal) present in the cytoplasm or on the surface of cells were counted. A minimum of 10 cells per treatment were counted and the data are expressed as the average number of B5 stained virions per cell. The error bar depicts the standard deviation and *P* value <0.05 is indicated (*t*-test).

Given that reduced RAB1A expression did not affect VACV entry or IMV production, but did demonstrate a substantial negative effect specifically on EEV production, we concentrated on the hypothesis that RAB1A depletion blocks EEV production through disruption of Golgi function. Our initial question was whether RAB1A depletion resulted in disruption of the Golgi as expected (Haas et al., 2007). HeLa cells were transfected with siRNA targeting RAB1A or VP16 for 48 h before being fixed and permeabilised. Cells were then labelled with antibody raised against the Golgi marker GM130. Focal, perinuclear Golgi labelling could be clearly identified in VP16 siRNA transfected cells; however in cells treated with RAB1A siRNA the fluorescence was reduced and dispersed (Fig. 3A), consistent with disruption of Golgi due to impaired RAB1A function.

To identify the point at which EEV production is impacted by reduced levels of RAB1A, we assessed the number and location of IEV and CEV particles present in cells in which RAB1A is knocked down. We repeated the siRNA transfection of HeLa cells, then infected with VACV-A5L-EGFP for 8 h prior to fixation and permeabilisation. The cells were then treated with antibody directed against the viral protein B5, which is found in IEV and CEV but not IMV membranes (Engelstad et al., 1992). Numerous IMV and IEV/CEV particles were identified in VP16 siRNA transfected cells (Fig. 3B, left panels). The IMVs (green) were mainly aggregated in viral factories in perinuclear regions, whereas the B5-labelled virions (red) were scattered throughout the cytoplasm and occasionally visible as CEVs on tips of actin tails (Fig. 3C). Cells with reduced levels of RAB1A contained similar amounts of IMVs within punctate perinuclear viral factories; however very few red labelled virions (IEVs or CEVs) or actin tails were detected in these cells (Fig. 3B, right panels). Quantification of the number of IEV/CEVs present in multiple VP16 and RAB1A siRNA transfected cells revealed a significant reduction in cells with reduced levels of RAB1A (Fig. 3D). These findings are consistent with the reduction in viral plaque size identified earlier (Fig. 2E), and suggest a role for RAB1A in the processing of IMVs to become IEVs.

**Fig. 4:**
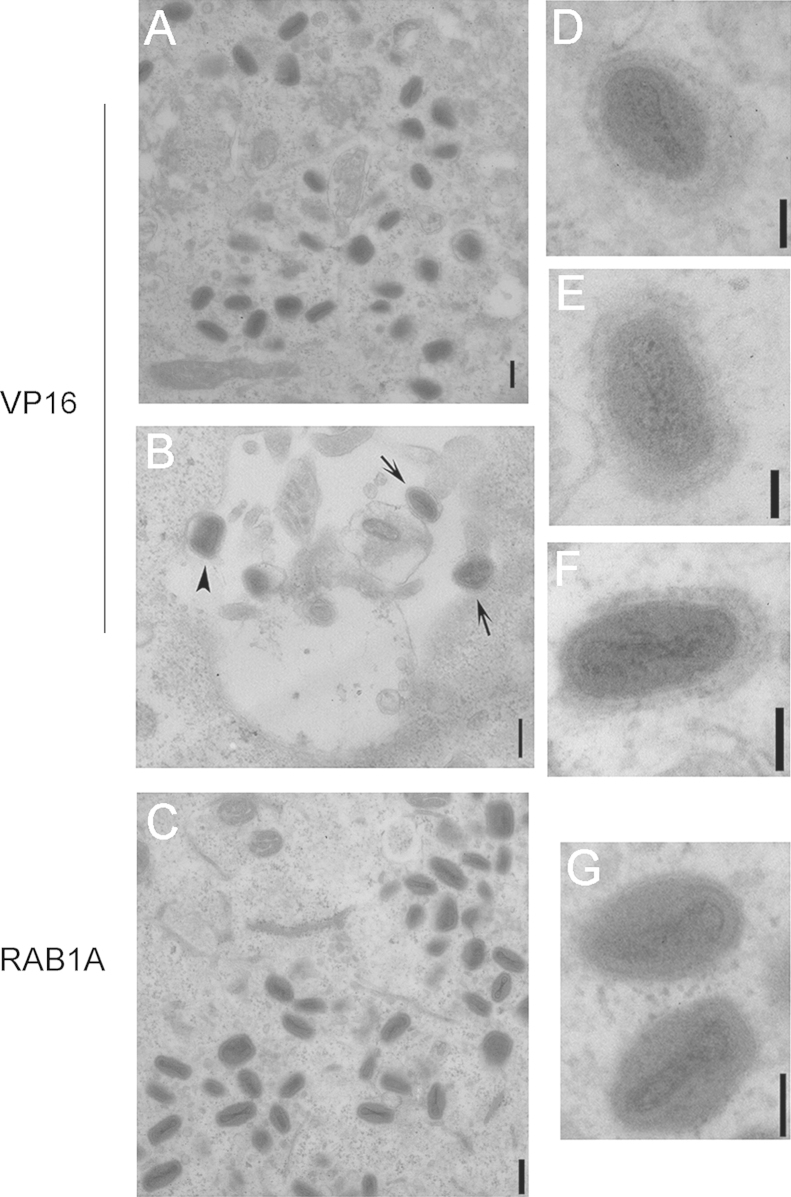
RAB1A knock down results in reduced IEV presence in a cell. HeLa cells were transfected with siRNA targeting VP16 or RAB1A for 48 h then infected with VACV at MOI=5 for 8 h before being fixed with 4% PFA, 2.5% gluteraldehyde in 0.2M PIPES and processed for TEM. (A, C) Images of cytoplasmic viral factories containing multiple IMVs. (B) Image of the surface of a cell with the arrowhead pointing to a CEV on the cell surface, and arrows pointing to two CEVs on the tips of actin tails. (D–F) High magnification images of IEVs within VP16 siRNA transfected cells. (G) High magnification image of two IMVs within a RAB1A siRNA transfected cell for comparison. Scale bars for images A–C=214 nm, for images D–G=88 nm.

In order to assess the abundance of IEVs directly, transmission electron microscopy was carried out on HeLa cells transfected with siRNA targeting RAB1A or VP16 and then infected with VACV WR for 8 h. Cytoplasmic viral factories were identified in cells transfected with either VP16 or RAB1A. These factories contained numerous IMVs (Fig. 4A and C). Within the cytoplasm of VP16 siRNA transfected cells we also detected multiple IEVs that were identifiable by their additional two membranes creating a well-defined shadow of irregular thickness around the virion core that added to the overall width of the particle (Fig. 4D–F). In addition occasional CEVs were noted on the surface of the VP16 siRNA transfected cells on the tips of actin tails (Fig. 4B). In contrast to the various virion forms present in VP16 siRNA transfected cells, the only mature virions present in RAB1A siRNA transfected cells were IMVs (Fig. 4C and G). No IEVs or CEVs or actin tails were identified in these cells. These results are consistent with the immunofluorescence results above and indicate that RAB1A loss does not influence the production of IMVs but does result in a reduction in the wrapping of IMVs to become IEVs.

## Discussion

RAB1A is known to act as a proviral host factor for a number of viruses including HIV, HCV and HSV-1. This work reveals that RAB1A is also required for optimal VACV replication and pinpoints the timing of RAB1A׳s mechanism of action to the transition from IMV to IEV. Onestep growth curves and viral protein expression profiles indicated that, despite its involvement in a wide range of cellular functions that contribute to VACV replication including integrin localisation and actin regulation, RAB1A had a negligible effect on early stages of viral replication with normal amounts of early and late proteins and IMVs produced in the absence of RAB1A (Fig. 2). The negative effect of RAB1A depletion on multicycle growth of VACV (Fig. 1A and C) is therefore likely due entirely to the loss of IEVs, CEVs and EEVs, emphasising the key role previously established for CEVs and EEVs in promoting rapid spread of VACV in cell culture (Blasco and Moss, 1991, 1992), reviewed by Smith et al. (2003).

Previous work used pharmacological agents and dominant-negative inhibitors to show that the wrapping of IMVs to form IEVs requires a functional ER to Golgi transport system. Cells treated with brefeldin A, which causes collapse of Golgi membrane stacks, did not influence IMV production but did reduce EEV production (Ulaeto et al., 1995). Similarly, infection of cells with a recombinant VACV strain expressing a dominant-negative Sar1 protein (which is required for initiation of ER to Golgi transport) resulted in inhibition of EEV production but did not affect IMV production (Husain and Moss, 2003). RAB1A plays a crucial role in later stages of ER to Golgi transport by facilitating the fusion of COPII coated vesicles with the cis face of the Golgi, and our results show that RAB1A, like Sar1, is required for IEV, CEV and EEV but not IMV production. This is consistent with ER to Golgi vesicle trafficking being the mechanism of function of RAB1A in the VACV lifecycle. It also highlights ER to Golgi transport as a possible target for novel antipoxviral therapies, or a means by which to modulate poxviral replication.

Investigation of the role of cellular regulators of vesicle trafficking in poxvirus morphogenesis is a powerful tool for dissecting the complex cell–host interactions required for VACV virion formation. Further investigations into interactions between poxviruses and components of the vesicle trafficking system, such as the regulatory Rab proteins, are likely to shed further light on poxvirus morphogenesis and are worthy of further study.

## Materials and methods

### Cells, viruses and antibodies

African green monkey kidney epithelioid cells (BS-C-1), and human cervix carcinoma epithelioid cells (HeLa) were grown in Dulbecco׳s modified Eagle׳s medium (DMEM) (Life Technologies) containing 50 IU/ml penicillin, 50 μg/ml streptomycin (Sigma) and 10% foetal bovine serum (FBS) (Life Technologies). Cells were incubated at 37 °C in a 5% CO_2_ incubator. VACV strain Western Reserve (VACV) and VACV-A5L-EGFP (Carter et al., 2003) were used in this work. The anti-A46 antibody was a kind gift from Andrew Bowie (Stack et al., 2005), the anti-D8 antibody a kind gift from Geoffrey Smith (Parkinson and Smith, 1994). Other antibodies used were anti-RAB1A (ab97956, Abcam), anti-GM130 (610822, BD Pharmingen), and anti-B5 (NR-556, BeiResources).

### siRNA knockdown of RAB1A

siRNA transfection was carried out in HeLa cells as described previously (Beard et al., 2014). RAB1A SMARTpool or deconvoluted RAB1A siRNAs, PRK AB1, VP16, or RSCF siRNA (Dharmacon/Thermo Scientific) were added to a final concentration of 25 nM and the cells were then incubated at 37 °C, 5% CO_2_ for 48 h before further experimental steps were carried out. Sequences of siRNAs used were as follows: RAB1A DC1 – GAACAAUCACCUCCAGUUA, DC2 – CAAUCAAGCUUCAAAUAUG, DC3 – GGAAACCAGUGCUAAGAAU, DC4 – CAGCAUGAAUCCCGAAUAU; PRK AB1– CAGAAGCCACAAUAACUUU; VP16 – GGGCGAAGUUGGACUCGUAUU.

### Western blotting and growth curves

The methods used were described previously (Haga et al., 2014). Briefly for single-step (or onestep) growth curves, HeLa cells were transfected with the siRNA indicated for 48 h before being infected with VACV at an MOI of 10 for 1 h at 37 °C. The cells were then washed with medium and incubated for the times indicated. Supernatants were collected and centrifuged at low speed to remove cell debris, then incubated with Rb168 antibody (Law and Smith, 2001) for 1 h at 37 °C in order to neutralise IMV particles. The titres of the virus present in the supernatants were determined by plaque assay on BS-C-1 cells. The titre of the virus in the cellular fraction was determined by collecting the cells and subjecting them to three freeze/thaw cycles and sonication before titering on BS-C-1 cells. Four biological replicates were performed. For the multistep growth curves, HeLa cells were transfected with the siRNA indicated for 48 h before being infected at MOI of 0.01 for 1 h at 37 °C. At the times indicated the supernatant and cellular fraction were combined by scraping the cells into the supernatant. The combined supernatant and cellular fraction was then frozen and thawed three times and sonicated before viral titres were determined by plaque assay on BS-C-1 cells. Five biological replicates were performed.

### Measurement of comet assays and plaque diameter

The technique was adapted from Blasco and Moss (1992). BS-C-1 cell monolayers were transfected with non-targeting siRNA (VP16) or siRNA targeting RAB1A, as described above. After 48 h the monolayers were infected with dilutions of VACV-WR. After 1 h at 37 °C, the virus inoculum was removed, fresh DMEM was added, and the incubation continued at 37 °C. After a further 5 h the medium was replaced with DMEM containing 1 μg/ml trypsin. The incubation then proceeded at 37 °C until 72 h postinfection, when monolayers were stained with crystal violet. Images of comet tails and plaques were taken using a Zeiss Axiovert 40 CFL inverted microscope and a Canon Powershot A640 camera. The length of the comet tails and diameter of the viral plaques were determined using Adobe Photoshop image software.

### Confocal microscopy

HeLa cells were seeded onto glass coverslips in a 6-well plate and incubated for 24 h at 37 °C before transfection with siRNA. After a further 48 h incubation cells were infected with VACV-A5L-EGFP (MOI=5) for 1 h at 37 °C. Inocula were removed and cells overlaid with 2.5% DMEM. At 8 hpi the media was removed, cells were washed gently thrice with ice cold PBS, then fixed with 10% neutral buffered formalin. Cells were then washed again thrice with PBS and permeabilised for 5 min with 0.01% Triton X-100. The samples were then incubated for 1 h at RT with antibody directed against B5 (1:500) or GM130 (1:500), followed by three washes in PBS with 2% FBS. The coverslips were then incubated for 1h at RT with AlexaFluor conjugated anti-rabbit or anti-mouse antibody (Life Technologies) and, where indicated, AlexaFluor conjugated phalloidin (Life Technologies), followed by three further washes in PBS. Finally, the coverslips were mounted with ProLong^®^ Gold with DAPI (Molecular Probes). Images were acquired using a Zeiss LSM 710 confocal microscope and Zen 2011 software (Zeiss).

### Electron microscopy

HeLa cells were seeded onto glass coverslips, tranfected as described above, incubated for 48 h at 37 °C and then infected with VACV (MOI=5). At 8 hpi cells were fixed in 4% PFA, 2.5% gluteraldehyde in 0.2 M PIPES, and washed in 0.2 M PIPES. Cells were post-fixed in 1% osmium tetroxide, washed in dH_2_O, dehydrated through graded ethanols into propylene oxide and then infiltrated with Durcupan resin through propylene oxide/Durcupan steps. Cells in Durcupan were then placed in moulds and baked at 60 °C overnight. Ultra-thin sections (Leica Ultramicrotome) were stained with uranyl acetate and lead citrate and viewed using a JEOL 1200EX transmission electron microscope.

### Statistical analysis

Analyses of fluorescence levels in multistep and onestep growth curves, comet tail length, plaque diameter, and the number of IEV/CEV in a cell were carried out using a Student׳s *t*-test. Log transformations of the titration data from multistep and onestep growth curves were analysed using a repeated measures mixed model (Brown and Prescott, 2006). Each model fitted: treatment, time, and the treatment×time interaction as fixed effects; and replicate, treatment×replicate interaction and replicate×time interaction as random effects. The inclusion of the replicate effects and their interactions as random allowed variation occurring between the replicates to be taken into account in the confidence intervals and the statistical tests used to compare treatments at each time point.

## Funding source

The Roslin Institute receives Institute Strategic Grant funding from the Biotechnology and Biological Sciences Research Council (BBSRC).
